# *THOP1* Is Entailed in a Genetic Fingerprint Associated with Late-Onset Alzheimer’s Disease

**DOI:** 10.3390/biom15030337

**Published:** 2025-02-26

**Authors:** Sharlee Climer

**Affiliations:** Alzheimer’s Disease Neuroimaging Initiative (ADNI), University of Missouri-St. Louis, St. Louis, MO 63121, USA; climer@umsl.edu

**Keywords:** THOP1, Alzheimer’s disease, genetic risk factor, haplotype, BlocBuster

## Abstract

In a systematic explorative study of genetic patterns on chromosome 19, we discovered a pattern comprising 23 SNP alleles that is significantly associated with late-onset Alzheimer’s disease (AD). This association was validated using two independent datasets. The pattern includes *thimet oligopeptidase* (*THOP1*), which has a long and disputatious relationship with AD. It also spans *solute carrier family 39 member 3* (*SLC39A3*) and *small glutamine-rich tetratricopeptide repeat co-chaperone alpha* (*SGTA*) and is upstream from *DIRAS family GTPase 1* (*DIRAS1*). We utilized population data to observe the frequencies of this genetic pattern for 11 different ancestries and noted that it is highly common for Europeans and relatively infrequent for Africans. This research provides a distinct genetic signature for AD risk, as well as insights into the complicated relationship between this disease and *THOP1*.

## 1. Introduction

Thimet oligopeptidase (THOP1), also known as EP24.15, MP24.15, and EC 3.4.24.15, belongs to the M3 family of zinc-dependent endopeptidases [1]. It moves between the cytoplasm and nucleus of neurons and glial cells and is secreted into extracellular space via multiple mechanisms [2,3,4,5,6]. Its function in major histocompatibility class I antigen presentation has been well studied, and more recently discovered roles in hyperlipidic diet-induced obesity, non-alcoholic liver steatosis, and insulin resistance are under investigation [7]. Due to the location of its active site at the bottom of a deep channel, THOP1 only cleaves peptides up to about 22 amino acids in length [7,8] but includes impactful substrates such as opioid peptides, bradykinin, somatostatin, neurotensin, angiotensin I, and gonadotrophin-releasing hormone (GnRH) [9,10,11,12].

While knockout of THOP1 does not affect viability, it lowers attention and memory retention and leads to depressive-like behavior [1]. In a sleep-depravation study of rat brains, THOP1 was shown to be dysregulated during depravation and recovery [13]. Based on the study of THOP1^−/−^ mice, it was shown that nearly 400 genes were differentially expressed in hippocampus samples, and it has been suggested that THOP1 may regulate at the transcriptional or post-transcriptional level via currently unknown mechanisms [1,7].

THOP1 has a long and tumultuous history with late-onset Alzheimer’s disease (AD) dating from the 1990s. Due to the discovery that it was capable of cleaving the amyloid precursor protein (APP) beta-secretase cleavage site in test peptides, it was a prime suspect for the production of sticky amyloid-beta peptides, which ultimately congregate into the amyloid plaques that are a hallmark of AD. Several ensuing investigations explored this mechanism, ultimately resulting in the conclusion that it did not significantly alter amyloid-beta production [14,15]. However, Papastoitsis et al. demonstrated that THOP1 leads to the degradation of recombinant APP and produces 15-kDa amyloidogenic fragments [16]. Based on further experiments using healthy human neuroblastoma cells, in 1999 this same group hypothesized that THOP1 leads to the degradation, rather than generation, of amyloid-beta peptides, presumably by modulating serine proteases [17]. Meanwhile, in 1996, *THOP1* was connected with AD due to its linkage with the risk-associated region on chromosome 19q13.3 [18]. Two years later, the location was corrected to 19p13.3, thereby refuting this linkage [19]. The strong signal at 19q13.3 is due to variants of *APOE*, which currently has the strongest known genetic associations with AD.

In 2008, a comprehensive proteomics analysis by Pollio et al. using cultured cortical neurons demonstrated knockdown of THOP1-increased amyloid-beta toxicity, while overexpression was neuroprotective [20]. Furthermore, this group observed increased THOP1 expression in the brain tissue of an AD transgenic mouse model and co-localization with amyloid-beta plaques. They also reported significant elevation of THOP1 expression in human AD brain tissue. Based on these concordant findings, Pollio et al. hypothesize that THOP1 provides neuroprotection in the early stages of AD. A recent broad transcriptomic study of AD brains further corroborated this association, as THOP1 was shown to exhibit a neuroprotective role with a highly significant false discovery rate [21].

Within the last few years, THOP1 has emerged as a candidate cerebrospinal fluid (CSF) biomarker for AD. Campo et al. observed that it was among the top five differentially regulated between AD and non-AD dementias out of 665 proteins measured in 797 CSF samples [22], and they developed a panel of seven CSF biomarkers, including THOP1, to discriminate between Lewy body dementia and AD [23]. This group recently developed an antibody-based platform to measure CSF THOP1 with the aim of diagnosing AD at an early stage [24,25].

We encountered *THOP1* in an unbiased investigation of genetic patterns on chromosome 19 that are associated with AD. Haplotypes are patterns of nucleotides in close proximity along a chromosome that are passed together across time and space. These patterns hold more power to dictate the specific properties of proteins produced and the regulation of this production than single variants. General haplotype inference methods phase all provided genotypes within the region of interest into two haplotypes without regard for the ages or evolutionary impact of each mutation, thus forcing the inclusion of more recent and/or neutral mutations. Alternatively, our previously introduced software, BlocBuster, identifies partial haplotypes that include only highly correlated nucleotides, referred to as blocs [26,27]. BlocBuster is a network modeling strategy that overcomes the pitfalls of conventional network methods by leveraging an expanded network scaffolding and a unique correlation metric that anticipates the heterogeneity of the individuals [28]. Due to these unique features, our exploration of the International HapMap [29] data for 11 global populations revealed an intriguing pair of massive blocs with opposite variants encompassing gephyrin on human chromosome 14 [30]. Open source code for BlocBuster is available at https://www.cs.umsl.edu/~climer/blocBuster/code.html (accessed on 20 February 2025).

The aim of our research is to leverage BlocBuster for advancing our knowledge of genetic patterns associated with AD. Given the heterogeneous nature of AD, we hypothesized that a systematic BlocBuster analysis may uncover blocs that are associated with subtypes of this complex disease. We utilized the Alzheimer’s Disease Neuroimaging Initiative (ADNI) genotype data, and our investigation revealed a bloc comprising 23 SNP alleles spanning *THOP1* and its upstream region. This pattern is quite common in the ADNI individuals, with more than 60% of the normal controls being carriers, but extremely prevalent for AD cases. We validated this result in two separate trials of unseen data.

Being interested in the incidence of this pattern, we examined the HapMap data to determine ancestral frequencies. While it was not possible to evaluate the entire 23-SNP pattern due to differences in the platforms, it is patently clear that the risk pattern is more widespread for European ancestries and less common for African populations.

THOP1′s association with AD has been enigmatic for several decades, and the presented research provides explicit details of genetic variants in high linkage that are significantly associated with this perplexing disease.

## 2. Materials and Methods

**AD data**: We analyzed data for chromosome 19 provided in the Alzheimer’s Disease Neuroimaging Initiative (ADNI) database (https://adni.loni.usc.edu (accessed on 20 February 2025)). The ADNI was launched in 2003 as a public–private partnership, led by Principal Investigator Michael W. Weiner, MD. The primary goal of ADNI has been to test whether serial magnetic resonance imaging (MRI), positron emission tomography (PET), other biological markers, and clinical and neuropsychological assessment can be combined to measure the progression of mild cognitive impairment (MCI) and early stages of AD. For up-to-date information, see www.adni-info.org (accessed on 20 February 2025).

WGS_Omni2.5M_20140220.zip containing genotypes for 812 individuals was downloaded, and 47,527 SNPs on chromosome 19 were extracted. No individual had more than 1% missing in the original data. We removed SNPs with more than 10% missing data. The data were split into Discovery (42 AD, 69 MCI, and 87 controls), Validation (18 AD and 37 controls), and Replication (38 AD and 63 controls) groups.

**HapMap data:** HapMap bulk data were downloaded from https://ftp.ncbi.nlm.nih.gov/hapmap/genotypes/2010-08_phaseII+III/forward/ (accessed on 20 February 2025). These data include genotypes for 11 populations: African Ancestry in SW USA [ASW], CEPH/Utah Collection with European ancestry [CEU], Han Chinese in Beijing, China [CHB], Chinese in Metropolitan Denver, CO, USA [CHD], Gujarati Indians in Houston, TX, USA [GIH], Japanese in Tokyo, Japan [JPT], Luhya in Webuye, Kenya [LWK], Mexican Ancestry in LA, CA, USA [MEX], Maasai in Kinyawa, Kenya [MKK], Toscani in Italia [TSI], and Yoruba in Ibadan, Nigeria [YRI]. The genotypes that were available for the 23 identified SNPs were extracted from each population. Individuals with more than 30% missing data for the extracted SNPs were excluded.

**BlocBuster:** Using BlocBuster, we built a network model using all AD, MCI, and control individuals (without labeling) in the Discovery dataset. Pairwise correlations were computed using the Custom Correlation Coefficient (CCC) [26,27]. CCC is robust for heterogeneous traits as it returns a vector of four values rather than a single scalar quantity. Nodes in the network represent SNP alleles, and edges represent pairwise correlations between alleles. We then extracted the highest weight edges such that the overall average node degree was equal to one. Breadth-First Search was used to extract clusters of inter-correlated SNP alleles that were completely isolated from the other nodes in the network. Each of these clusters represent a genetic pattern, and they were tested for association with AD.

## 3. Results

The BlocBuster network was built blindly from the Discovery data, without any information regarding AD, MCI, or normal control included. It had a strong community structure with 84.3% singleton nodes, for which no edge was adjacent. The connected components ranged in size from 2 to 135 nodes.

The genetic patterns corresponding to each component were tested for associations between the AD and normal control individuals in the Discovery data. Eleven patterns with more than 10 nodes had significant odds ratios and 95% confidence intervals and were tested on the Validation individuals. The most significant bloc from this trial comprised 23 SNP alleles and was tested using the Replication data. As shown in Table 1, this bloc has odds ratios and 95% confidence intervals of 2.632 [1.044, 6.631], 10.348 [1.238, 86.506], and 2.667 [0.964, 7.375], in the Discovery, Validation, and Replication datasets, respectively. Note that the 95% confidence interval for the Replication dataset spans slightly below 1.0, but due to the consistencies across all three datasets, this is likely due to the relatively small sample size rather than lack of genuine association.

**Figure 1:**
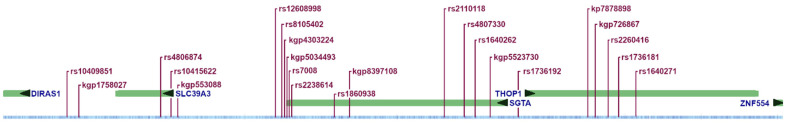
Relative genomic locations of the 23 single-nucleotide polymorphisms (SNPs) included in the associated pattern. See Supplementary Table S1 for precise locations.

The identified pattern is located on chromosome 19p13.3, beginning upstream from *DIRAS family GTPase 1* (*DIRAS1*) and spanning across *solute carrier family 39 member 3* (*SLC39A3*), *small glutamine rich tetratricopeptide repeat co-chaperone alpha* (*SGTA*), and *thimet oligopeptidase* (*THOP1*) (Table 2 and Figure 1). Annotations for the 23 SNPs are given in Supplementary Table S1.

The numbers of SNPs in the bloc that were included in the HapMap data ranged from 12 to 15 for the 11 populations (Table 3). The frequencies of carriers ranged from 0.136 for the LWK population to 0.879 for the CEU. In general, the European ancestries exhibited high frequencies, African populations exhibited low frequencies, and the other populations were between the two extremes. Genotypes for the extracted SNPs are given in Supplementary Tables S2–S12.

## 4. Discussion

The network model was created blindly using all individuals in the Discovery dataset, yet the strong linkage of the 23 SNPs was significantly associated with AD risk in all three datasets.

It is unclear how the identified genetic pattern impacts thimet oligopeptidase (THOP1) production. Most of the markers are upstream and may affect transcriptional regulation. Surprisingly, the other genes spanned by this bloc have intriguing relationships and may be more directly involved in the observed AD association.

THOP1 is a zinc-activated oligopeptidase, and solute carrier family 39 member 3 (SLC39A3), also known as ZIP3, transports zinc ions from extracellular space to the cytosol. While knocking out ZIP3 and another family member, ZIP1 provided neuroprotection for CA1 neurons; the silencing of only ZIP3 resulted in attenuated CA3 neuronal cell death in the mouse hippocampus [31,32].

Small glutamine-rich tetratricopeptide repeat co-chaperone alpha (SGTA) interacts with steroid receptor complexes and other chaperones, including heat shock proteins HSP70 and HSP90 [33]. In its role as a co-chaperone, it identifies misfolded proteins and has been shown to directly bind exposed hydrophobic residues in vitro and in vivo [34]. This behavior may be at the root of its colocalization with aberrant intracellular aggregates, including Huntington Disease and polyQ diseases [35]. SGTA is also indispensable for cell division [36]. In general, this protein behaves in complex manners with both tissue-specific and cell-specific functions [33].

DIRAS family GTPase 1 (DIRAS1) belongs to a subfamily of small Ras monomeric GTPases. It has been observed to act as a tumor suppressor, and down-regulation of this gene in several cancer types has been achieved via aberrant methylation [37]. Importantly, an epigenome-wide methylation study of entorhinal cortex in 337 human brains identified four differentially methylated genes associated with AD, one of which was DIRAS1 [38].

THOP1 is involved in many extracellular activities, such as the degradation of neuropeptides, including opioids, bradykinin, neurotensin, and gonadotropin-releasing hormone (GnRH) [39]. Interestingly, its secretion to extracellular space is unconventional as it lacks a signal peptide sequence [4]. It has been shown that interactions with 14-3-3 epsilon, a phosphoserine/threonine-scaffold protein, facilitates THOP1 secretion [4]. Correño et al. demonstrated that phosphorylation of THOP1 at Ser(644) by protein kinase A (PKA) increases 14-3-3 epsilon interaction, and the introduction of a point mutation S644A reduces it [4]. Future research is needed to delve into the 23-SNP genetic signature to pinpoint specific AD biological pathways, including the secretion and selection of substrates.

Genotypes for all 23 SNPs were not available for the HapMap individuals; however, at least half were provided for each population. It could be expected that this pattern is most prevalent in the European ancestral populations as the ADNI database is currently predominantly European, thereby generating a population-based bias. Yet it is quite surprising that the African populations exhibit such low frequencies. Note that inclusion of the missing genotypes cannot increase the frequencies and might lower them even further. Taken together, these results suggest that the use of primarily European ancestries in this study led to the discovery of an AD subtype that is not common for African ancestries. Future work is needed to evaluate the association of this bloc with AD for these populations.

## 5. Conclusions

The pattern presented in this manuscript provides insights into the pathophysiology of AD as it provides a high-dimensional genetic variation comprising 23 distinct SNP alleles that are strongly associated with this enigmatic disease. Future work using CRISPR and/or animal models, as well as the quantification of affected downstream products, including RNA and proteins, holds potential for revealing underlying mechanisms and causal relationships between this precise genetic feature and AD. More broadly, this manuscript exemplifies a research strategy for systematically exposing high-dimensional genetic patterns associated with complex phenotypes.

## Figures and Tables

**Table 1:** Results for the 23-SNP pattern for the three trials. Numbers and percentages of individuals, odds ratios, and odds ratio 95% confidence intervals are shown for late-onset Alzheimer’s disease (AD) cases and normal controls.

	AD Cases	Normal Controls	Odds Ratio	95% CI
**Discovery**	35 (83.33%)	57 (65.52%)	2.632	[1.044, 6.631]
**Validation**	17 (94.44%)	23 (62.16%)	10.348	[1.238, 86.506]
**Replication**	32 (84.21%)	42 (66.67%)	2.667	[0.964, 7.375]

**Table 2:** Single-nucleotide polymorphism (SNP) IDs and alleles for the 23-SNP genetic pattern.

SNP ID	Allele	SNP ID	Allele
rs10409851	A	kgp8397108	A
kgp1758027	C	rs2110118	T
rs4806874	A	rs4807330	G
rs10415622	A	rs1640262	T
kgp553088	C	kgp5523730	A
rs12608998	G	rs1736192	G
rs8105402	C	kgp7878898	G
kgp4303224	G	kgp726867	T
kgp5034493	C	rs2260416	G
rs7008	A	rs1736181	C
rs2238614	G	rs1640271	C
rs1860938	A		

**Table 3:** Results for HapMap individuals: African Ancestry in SW USA [ASW], CEPH/Utah Collection with European ancestry [CEU], Han Chinese in Beijing, China [CHB], Chinese in Metropolitan Denver, CO, USA [CHD], Gujarati Indians in Houston, TX, USA [GIH], Japanese in Tokyo, Japan [JPT], Luhya in Webuye, Kenya [LWK], Mexican Ancestry in LA, CA, USA [MEX], Maasai in Kinyawa, Kenya [MKK], Toscani in Italia [TSI], and Yoruba in Ibadan, Nigeria [YRI]. The number of single-nucleotide polymorphisms (SNPs) in the 23-SNP pattern that were genotyped in the HapMap data and the frequencies of carriers for the given patterns are shown for each of the 11 populations. The specific SNPs included for each population are given in Supplementary Tables S2–S12.

Population	# SNPs	Carriers Freq
ASW	12	0.356
CEU	15	0.879
CHB	14	0.504
CHD	13	0.468
GIH	13	0.752
JPT	14	0.566
LWK	13	0.136
MEX	13	0.826
MKK	13	0.402
TSI	13	0.775
YRI	14	0.201

## Data Availability

Data used in the preparation of this article were obtained from the Alzheimer’s Disease Neuroimaging Initiative (ADNI) database (adni.loni.usc.edu). As such, the investigators within the ADNI contributed to the design and implementation of ADNI and/or provided data but did not participate in the analysis or writing of this report. A complete listing of ADNI investigators can be found at http://adni.loni.usc.edu/wp-content/uploads/how_to_apply/ADNI_Acknowledgement_List.pdf (accessed 15 September 2024).

## Supplementary Materials

The following supporting information can be downloaded at: https://www.mdpi.com/article/10.3390/biom15030337/s1, Table S1 Annotations for the 23 SNPs in the AD risk pattern; Table S2 Genotypes for available SNPs corresponding to the 23-SNP pattern for the ASW individuals in the HapMap data; Table S3 Genotypes for available SNPs corresponding to the 23-SNP pattern for the CEU individuals in the HapMap data; Table S4 Genotypes for available SNPs corresponding to the 23-SNP pattern for the CHB individuals in the HapMap data; Table S5 Genotypes for available SNPs corresponding to the 23-SNP pattern for the CHD individuals in the HapMap data; Table S6 Genotypes for available SNPs corresponding to the 23-SNP pattern for the GIH individuals in the HapMap data; Table S7 Genotypes for available SNPs corresponding to the 23-SNP pattern for the JPT individuals in the HapMap data; Table S8 Genotypes for available SNPs corresponding to the 23-SNP pattern for the LWK individuals in the HapMap data; Table S9 Genotypes for available SNPs corresponding to the 23-SNP pattern for the MEX individuals in the HapMap data; Table S10 Genotypes for available SNPs corresponding to the 23-SNP pattern for the MKK individuals in the HapMap data; Table S11 Genotypes for available SNPs corresponding to the 23-SNP pattern for the TSI individuals in the HapMap data; Table S12 Genotypes for available SNPs corresponding to the 23-SNP pattern for the YRI individuals in the HapMap data.
